# Supplementary material for: L amino acid transporter structure and molecular bases for the asymmetry of substrate interaction
Source: Nat Commun. 2019 Apr 18;10:1807. doi: 10.1038/s41467-019-09837-z (PMC6472337; doi:10.1038/s41467-019-09837-z)
Supplement: Supplementary file 1 — Supplementary Information [file 41467_2019_9837_MOESM1_ESM.pdf]

L Amino acid Transporter structure and molecular bases for the asymmetry of substrate interaction

Errasti-Murugarren et al.

Supplementary Information

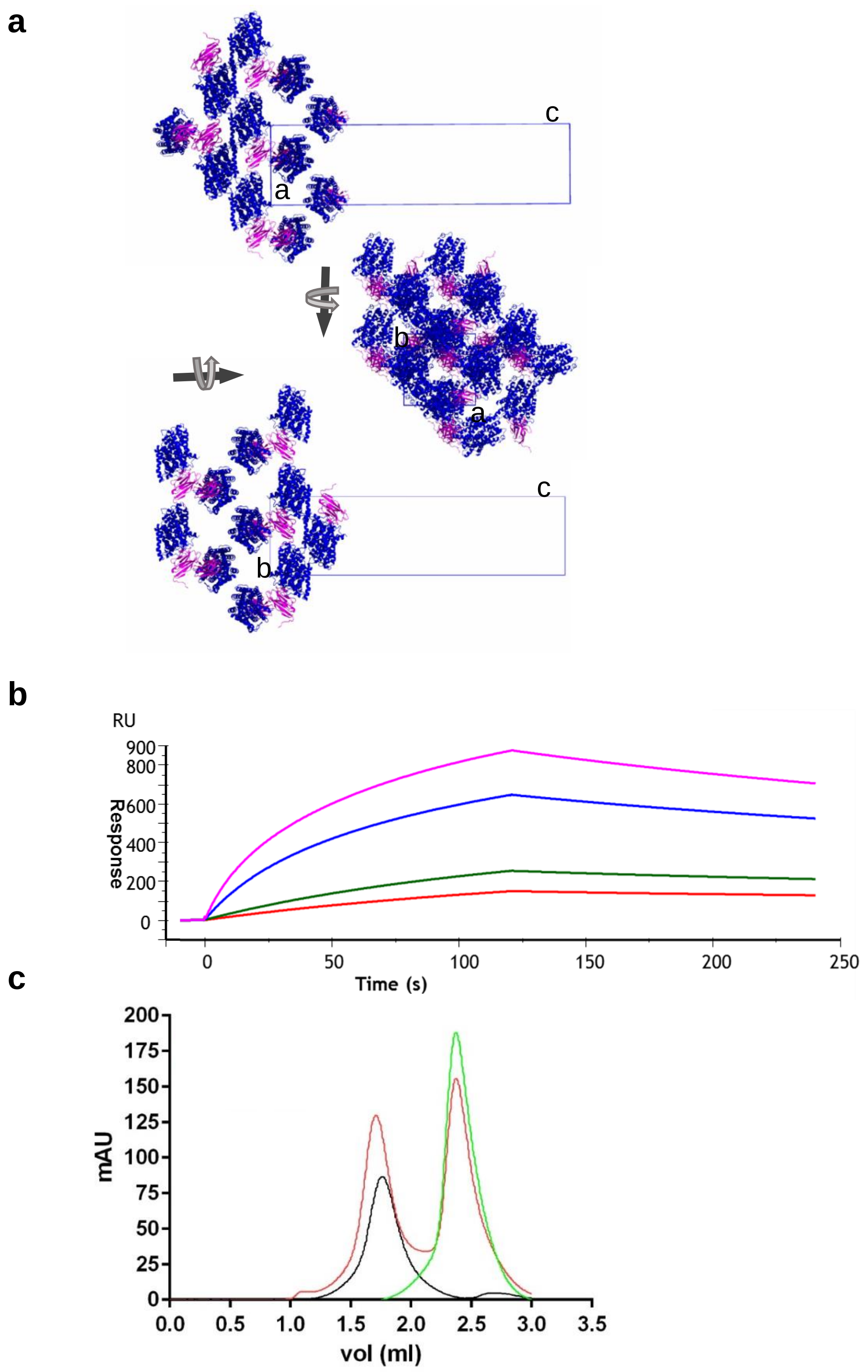

Supplementary Figure 1. Nb74 interaction with BasC. a) Crystal packing showing the unit cell. Most of the crystal contacts are provided by Nb74 (magenta). There is one molecule of BasC (blue) and Nb74 per unit cell. **b**) Surface plasmon resonance of Nb74–BasC interaction. K_a, K_d and K_D are $3.93 \cdot 10^5$ Ms^{-1}, $1.52 \cdot 10^{-3}$ s^{-1} and 3.87 nM, respectively. **c**) Analytical size exclusion chromatography (SEC) showing the formation of the BasC-Nb74 complex, as indicated. The Nb74 peak (green) decreases the amplitude when the two proteins are mixed before SEC (red). The BasC peak (gray) increases in size and amplitude when the two proteins are mixed before SEC.

a

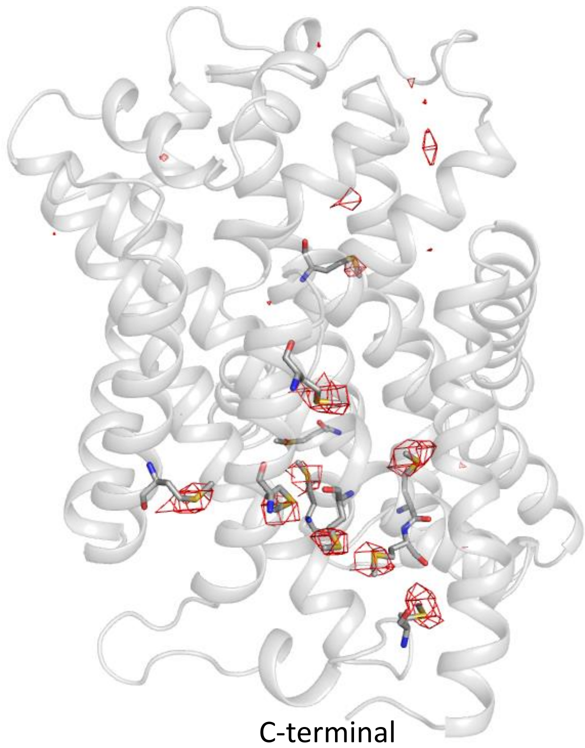

b

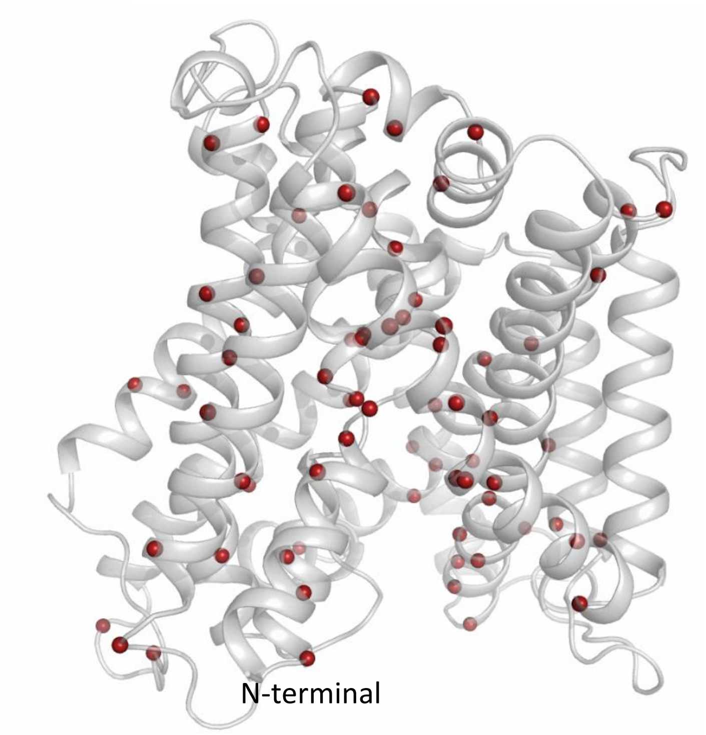

Supplementary Figure 2. a) Anomalous signal of SeMet fits with 10 out of 11 methionine positions (only lacking N-terminal Met) in the model of the BasC apo structure. Cartoon representation of the BasC structure showing methionine residues as sticks and Map of FDANO-F 3,0 sigma depicted in red. **b**) BasC structure cartoon with red spheres in the BasC residues homologous to the residues with missense mutations causing disease in human LATs.

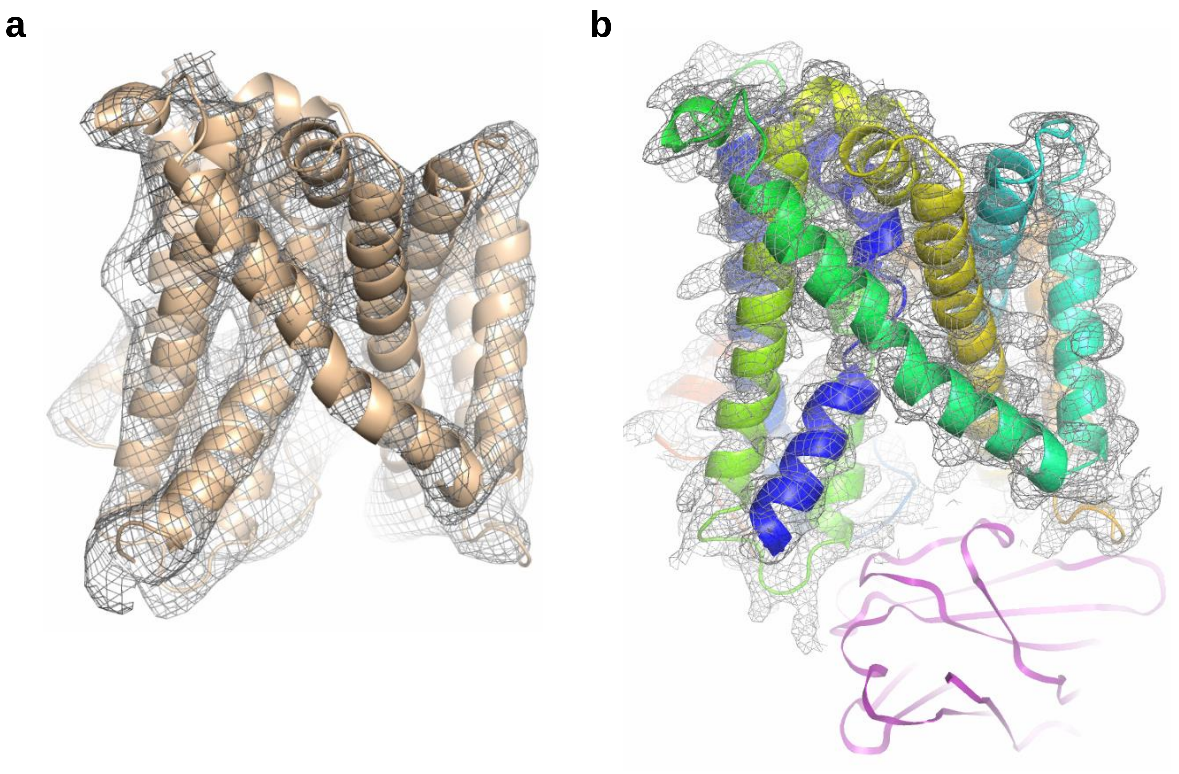

Supplementary Figure 3. Comparison of the crystal structures of BasC apo without Nb74 obtained with HiLiDe in DDM at 7.2 Å (cartoon colored in golden) **a)**, and of BasC apo in complex with Nb74 at 2.9 Å (BasC cartoon colored in rainbow and Nb74 in magenta) **b)**. Both structures with the 2Fo-Fc electron density map (gray) correspond to non-occluded inward-facing conformation.

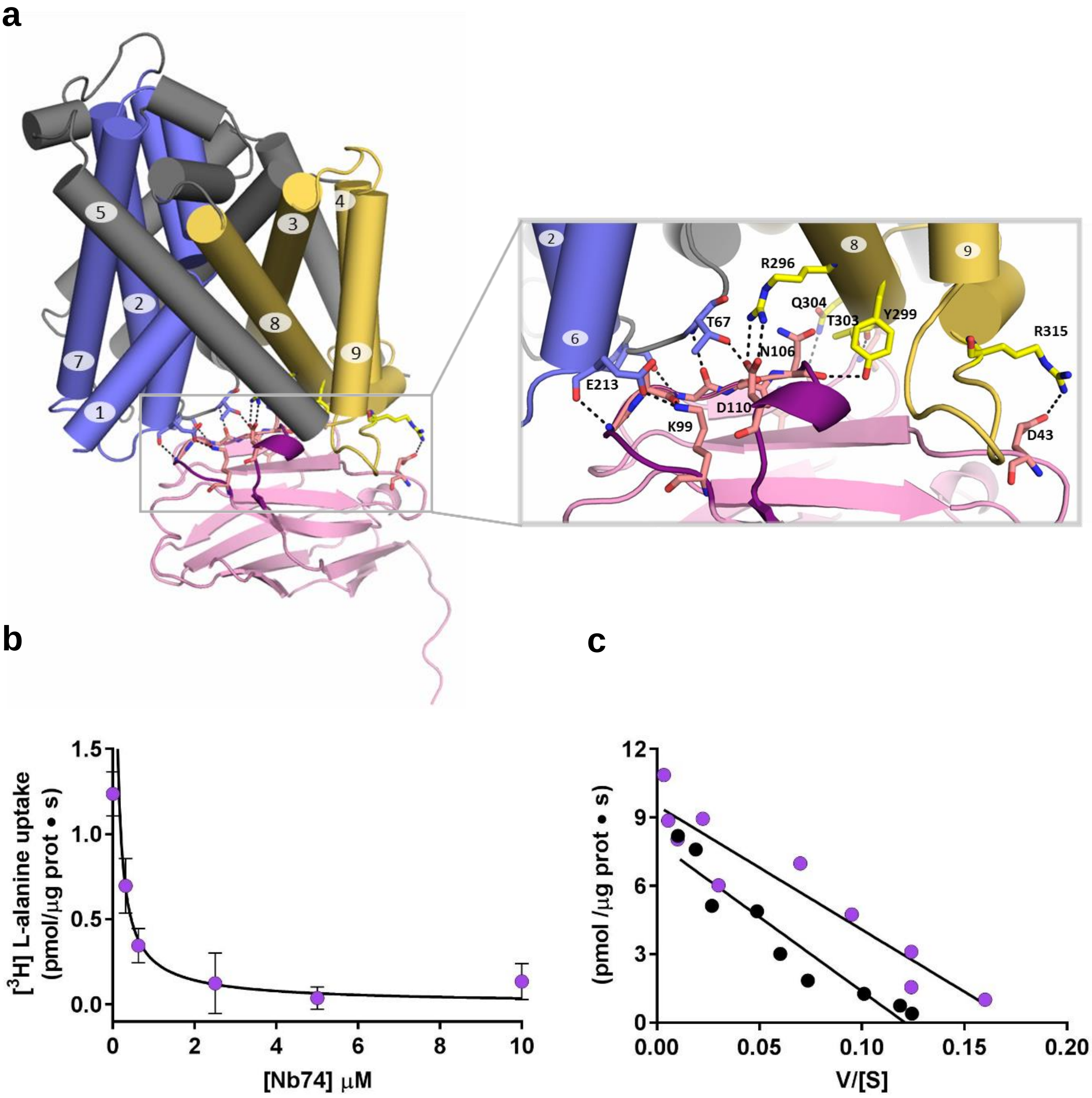

Supplementary Figure 4. Nb74 interacts with residues within the bundle and the hash domains (blue and yellow respectively) inhibiting BasC transport activity. Nb74 is shown in magenta. Extensive hydrogen bonding was found between the side chains and backbones of the complementary determining region 3 (CDR3) of Nb74 and BasC, as well as of two salt-bridges formed by Asp 110 with Arg 296 in CDR3 and BasC TM8, and by Lys 99 with Glu 213 in CDR3 and TM6b respectively. Additionally, Glu 43 in framework region (FR) 2 and Tyr 60 and Asp 62 in FR3 from Nb74 are involved in direct hydrogen bonding with Arg 315 in TM9 and Gln 304 and Thr 303 in intracellular loop 4 of BasC, respectively. Finally, Tyr 299 in TM8 and Thr 67 in intracellular loop 1 are also in contact with Nb74 through a hydrogen bond with Asn 106, Ala 104 and Asp 110 in CDR3, respectively. Interacting residues are designated with the one letter code and the number in the polypeptide chain. **b)** Inhibition dose-response curve of the 10 μM [^{3}H]L-alanine efflux in exchange with 4 mM L-alanine in BasC proteoliposomes by Nb74. Data (mean±s.e.m.) correspond to triplicates. **c)** Eadie-Hofstee linearization of the Michaelis-Menten plot from Figure 2C in the absence (black circles) and presence (magenta circles) of 5 μM NB74 in the external medium. [^{3}H]L-alanine concentrations up to 800 μM in the absence of Nb74 and up to 3200 μM in the presence of Nb74 were used for linearization. V/[S] is expressed in pmol μg prot^{-1} s^{-1} μM^{-1}). The slopes (-K_m) are similar, thereby indicating that determination of the extracellular K_m without the use of Nb74 and with a range of substrate concentration below 800 μM is a good estimation. Source data are provided as a Source Data file.

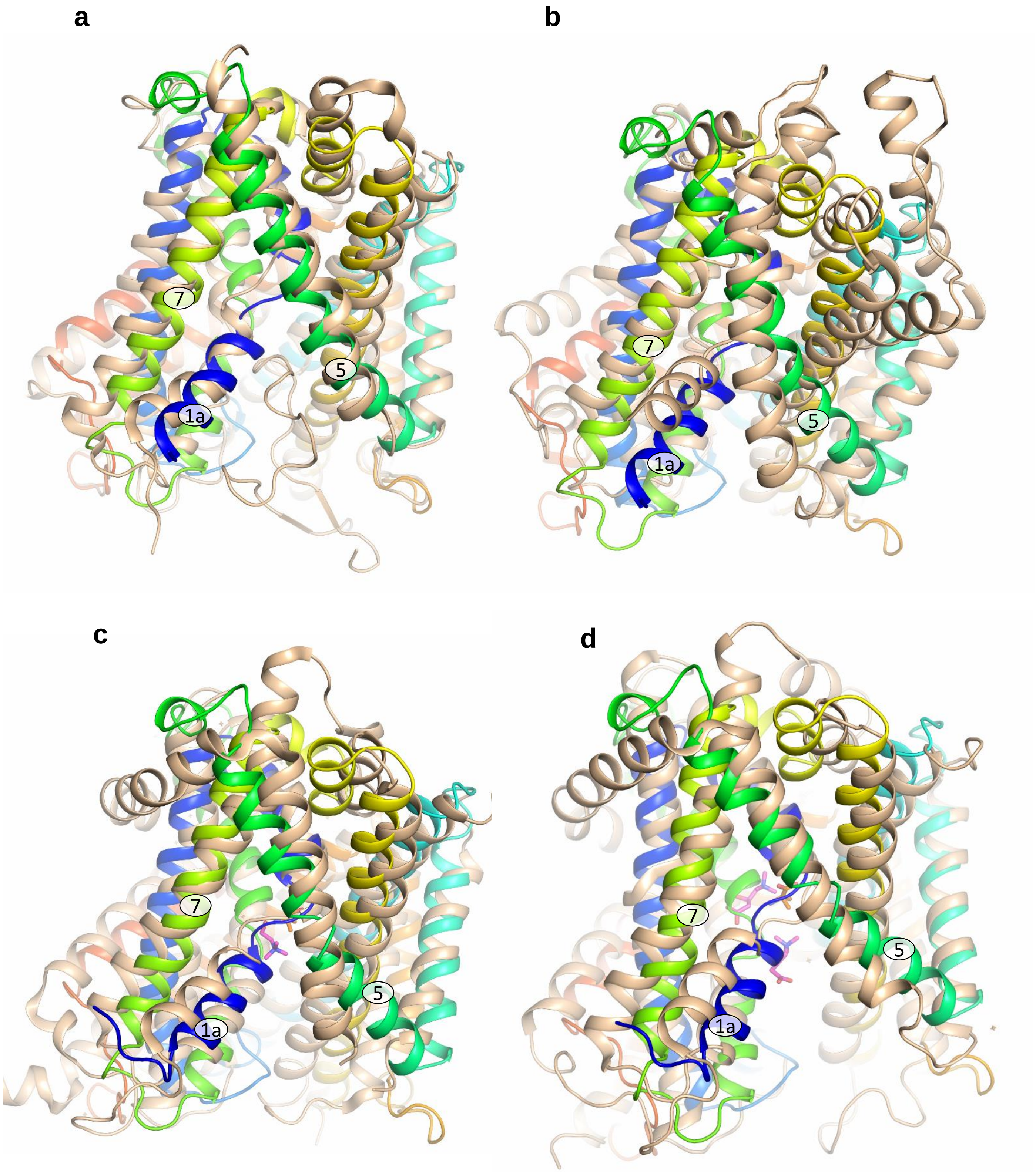

Supplementary Figure 5. Superimposition of BasC structures (rainbow) with some inward-facing APC superfamily transporters: a) GadC (4DJK)[1] and **b)** LeuT (3TT3)[2] with BasC apo (6F2G); and **c)** BetP (3P03)[3] and **d)** CaiT (3HFX)[4] with BasC holo (6F2W) (substrates in the binding site are represented as sticks).

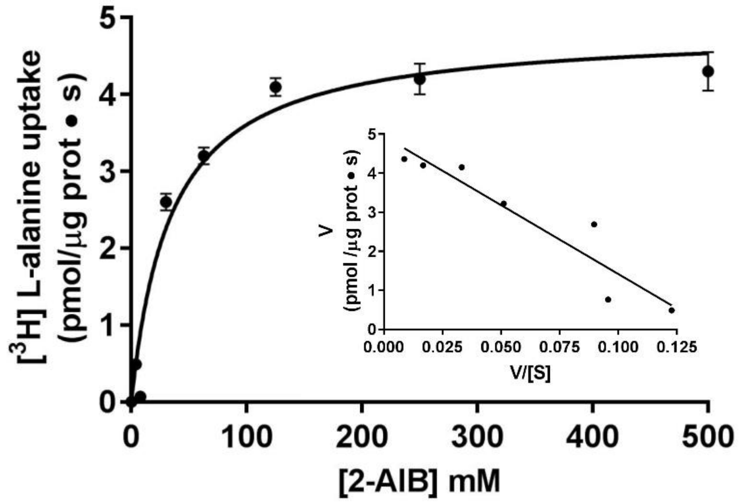

Supplementary Figure 6. Kinetics of 2-AIB transport by BasC. Kinetics of the exchange of 10 μM [^{3}H]L-alanine with a range of 2-AIB concentrations inside BasC PLs. The inset shows the Eady-Hofstee linearization used to estimate the cytoplasmic K_m (34.5±10 mM). Data correspond to the mean±s.e.m. of representative experiments. Source data are provided as a Source Data file.

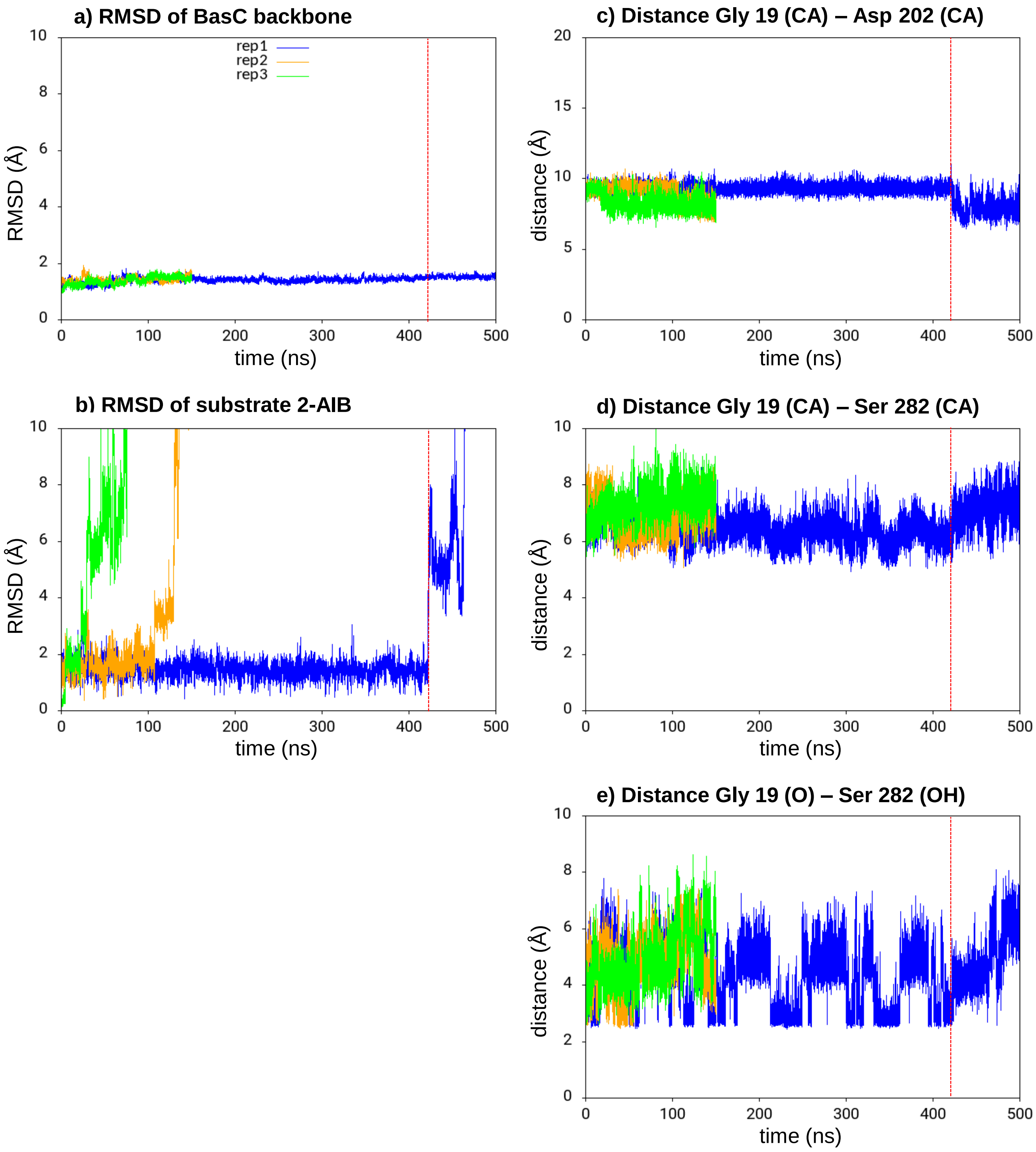
a) RMSD of BasC backbone
rep1
rep2
rep3
RMSD (Å)
time (ns)
b) RMSD of substrate 2-AIB
RMSD (Å)
time (ns)
c) Distance Gly 19 (CA) – Asp 202 (CA)
distance (Å)
time (ns)
d) Distance Gly 19 (CA) – Ser 282 (CA)
distance (Å)
time (ns)
e) Distance Gly 19 (O) – Ser 282 (OH)
distance (Å)
time (ns)

f) Distance 2-AIB (O) – Tyr 236 (OH)

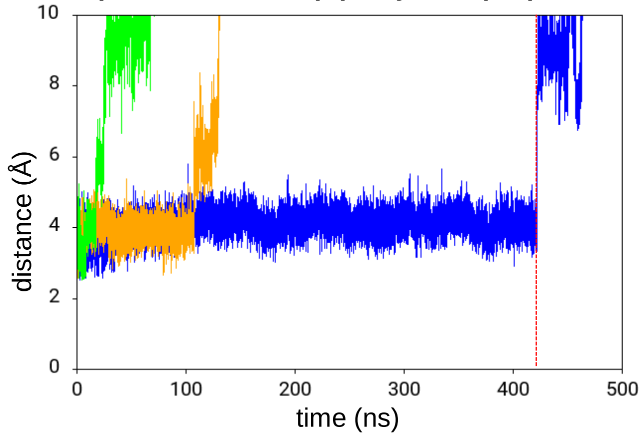

j) Distance 2-AIB (CA) – Lys 154 (NZ)

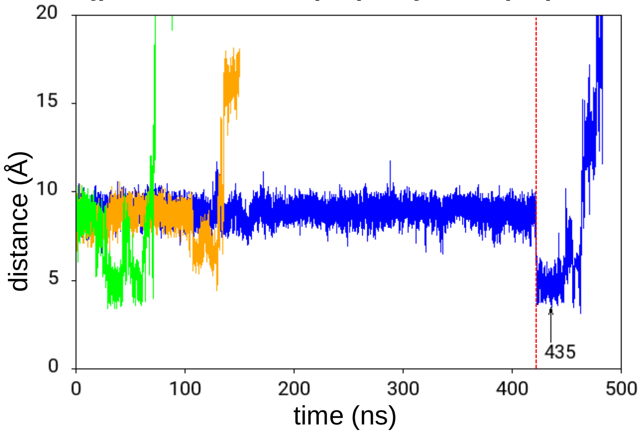

g) Distance 2-AIB (N) – Ala 200 (O)

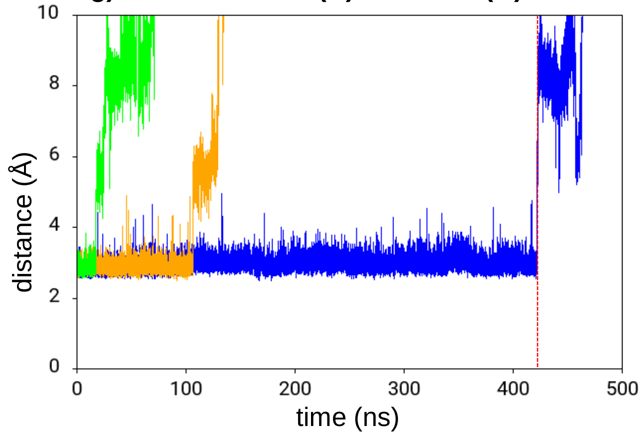

h) Distance Tyr 236 (OH) – Ala 200 (O)

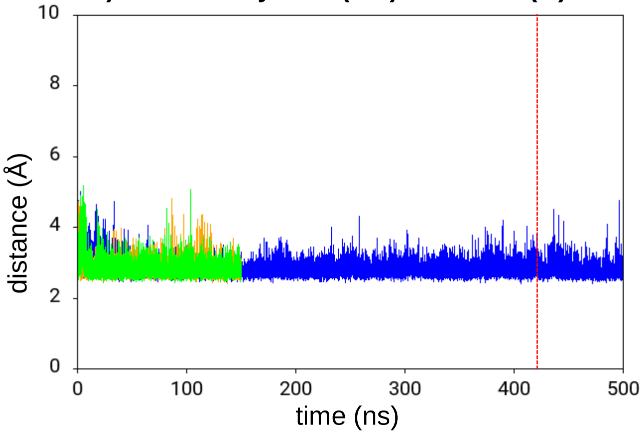

i) Distance Tyr 236 (OH) – Gly 19 (N)

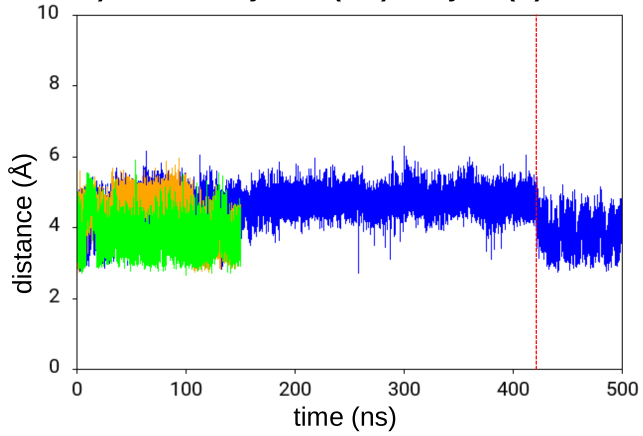

Supplementary Figure 7. Molecular dynamics analysis of BasC holo structure (PDB ID: 6F2W) without Nb74 bound. a) Evolution of root-mean-square deviation (RMSD) of the backbone atoms of BasC with respect to the initial structure along three different replicas (blue, green and orange). **b)** Evolution of RMSD of all atoms of the substrate 2-AIB along the same trajectories. **(c-j)** The rest of the panels show the evolution of the distances between the atoms shown in parentheses of the corresponding residues of BasC or the substrate 2-AIB, for the same trajectories. Atoms are named according to the PDB file: CA (C-α), O (carbonyl oxygen), OH (hydroxyl oxygen), N (amine nitrogen for 2-AIB and amide nitrogen for Gly 19), and NZ (ε amine nitrogen). The arrow in panel (j) indicates the time (435 ns) in replica 1 (blue) corresponding to the snapshot shown in Figure 6. Trajectories are depicted until a few nanoseconds after 2-AIB is released. This moment is indicated for replica 1 (in blue) as a vertical red dot line in all panels.

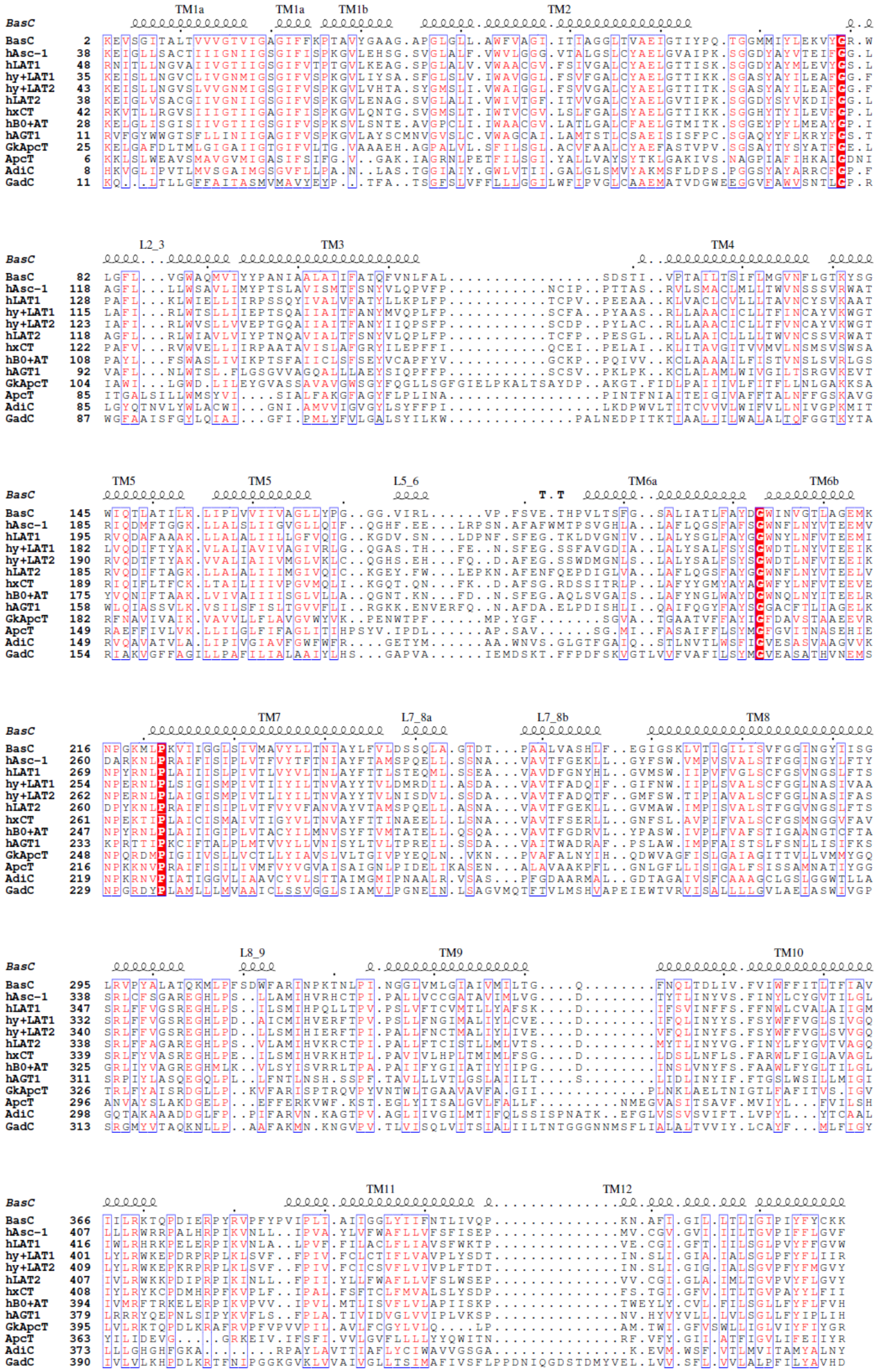

Supplementary Figure 8. BasC sequence alignment with human LATs and with GkApcT, ApcT AdiC and GadC. Multi sequence alignment was done with PSI/TM-Coffee server [5] and ENDscript server [6].

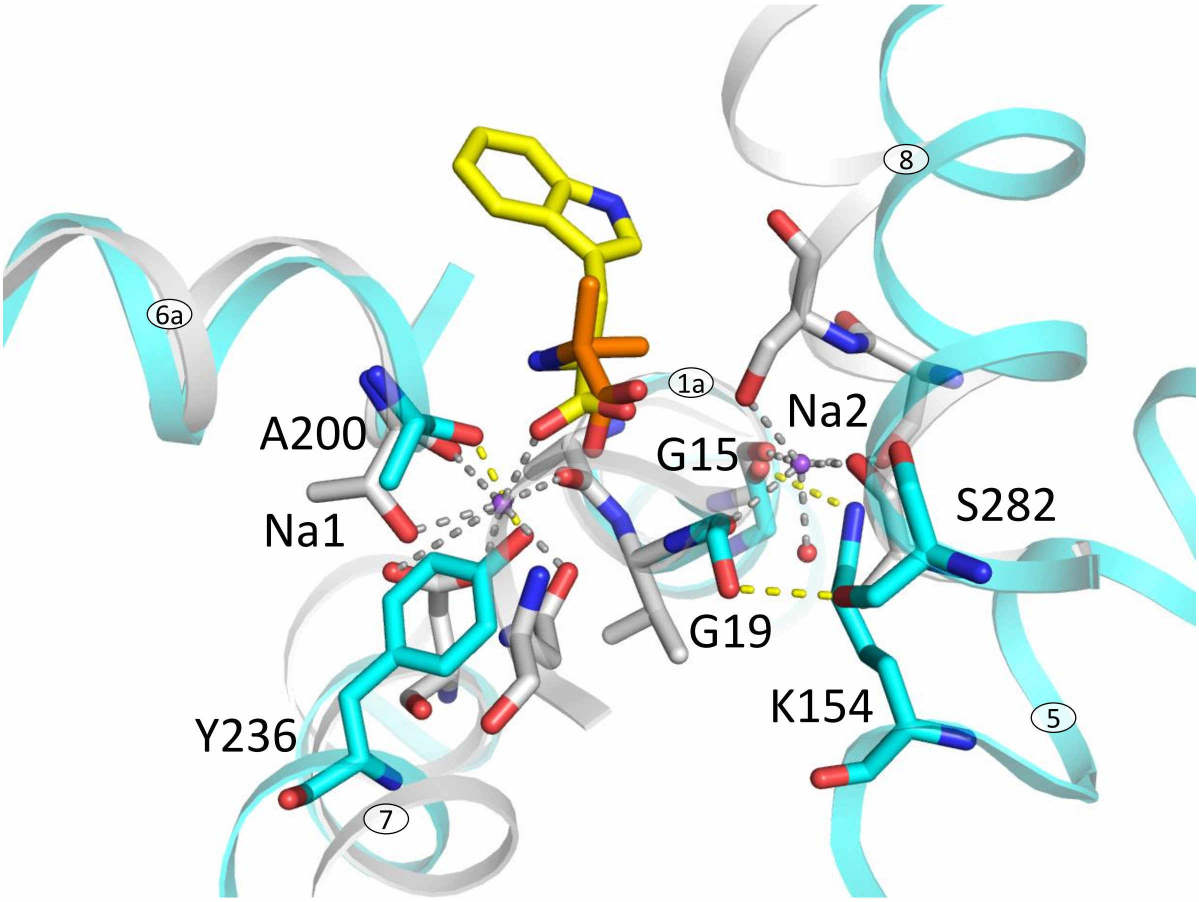

Supplementary Figure 9. BasC Tyr 236 and Lys 154 lie within the Na sites of sodium-dependent APC superfamily transporters. Superposition of 2-AIB (orange) bound BasC structure (cyan) with the MhsT structure (light gray, PDB ID: 4US4)[7] in occluded open to in conformation bound to two sodiums and one tryptophan (yellow) in the substrate-binding site. Depicted stick residues in BasC are labeled in the picture. Sodium coordinating residues are depicted as sticks in MhsT: A26 and N31 in TM1a, T231 in TM6a and D263 in TM7 in Na1 site and G24 and V27 in TM1a, A320, S323 and S324 in TM8 and a molecule of water in Na2 site.

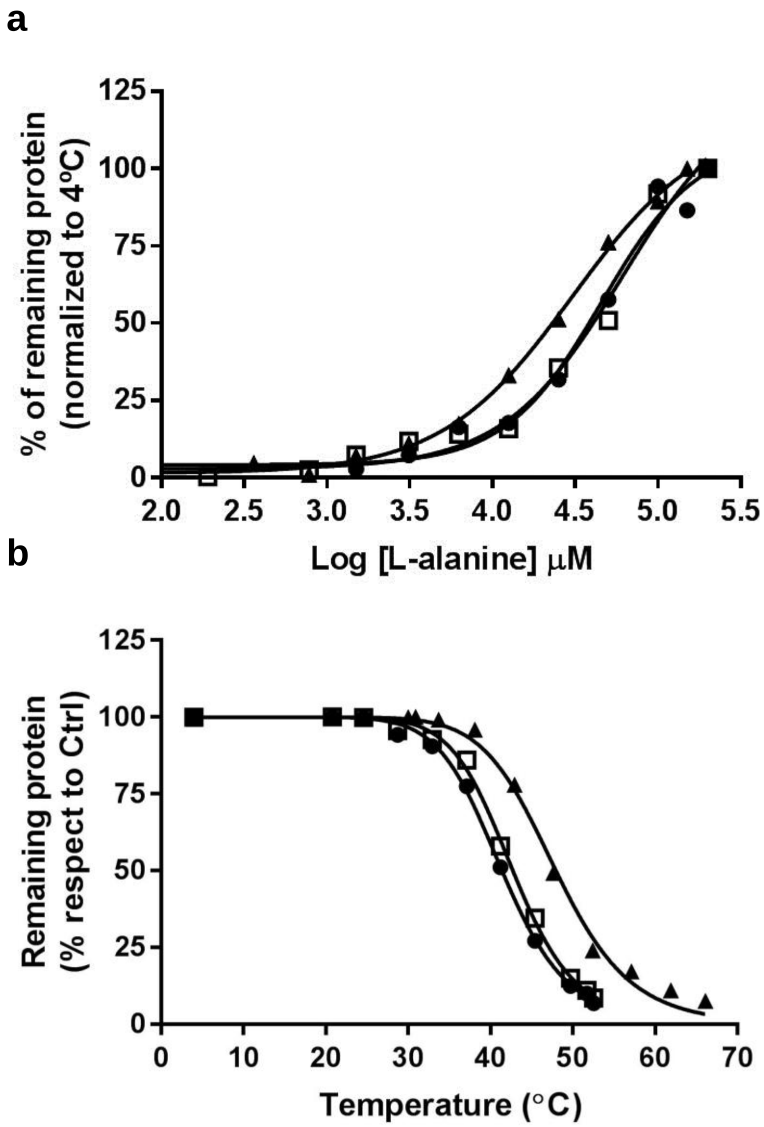

Supplementary Figure 10. L-alanine binding and thermostability in wild-type BasC, and in Y236F and K154A mutants. a) Estimation of K_D values for L-alanine in wild-type (WT) BasC (closed triangles) and Y236F (closed circles) and K154A (open squares) mutants. GFP-fused WT and mutant proteins were incubated for 10 min at the melting temperature (T_m) in the presence of a range of L-alanine concentrations (0–200 mM). The soluble protein (mean) remaining after 30 min ultracentrifugation at 186,000 × *g* was analyzed by size exclusion chromatography (SEC) and plotted *vs.* the $logarithm_{10}$ of L-alanine concentration. K_D values for WT BasC, Y236F and K154A were 34.5±2.1, 42.3±5, 61.4±7.3 mM, respectively. Student's t-test; WT *vs* Y236 not significant, WT *vs* K154A p=0.024. **b**) Estimation of the melting temperature (T_m) of WT BasC (closed triangles), and Y236F (closed circles) and K154A (open squares) mutants. GFP-fused WT and mutant proteins were incubated for 10 min at a range of temperatures (4–70°C, for wild type, 4–60°C for mutants). The soluble protein (mean) remaining after 30 min ultracentrifugation at 186,000 × *g* was analyzed by SEC, and data were normalized to the 4°C sample and plotted *vs.* temperature. T_m values for WT, Y236F and K154A were 47.9±0.8, 41.3±0.5 and 42.6±0.6°C, respectively. K_D and T_m are the mean±s.e.m. of three independent experiments. Source data are provided as a Source Data file.

	BasC apo PDB: 6F2G	BasC + 2-AIB: 6F2W	SeMet Basc
**Data collection**			
Space group	$P4_12_12$	$P4_12_12$	$P4_12_12$
Cell dimensions			
a, b, c (Å)	85.83, 85.83, 321.69	85.64, 85.64, 321.63	85.87, 85.87, 306.73
α,β,γ (°)	90.0, 90.0, 90.0	90.0, 90.0, 90.0	90.0, 90.0, 90.0
Wavelength (Å)	0.979260	0.979260	
Resolution (Å)	2.92	3.40	4.32
R_{merge}	0.058 (0.885)	0.097 (1.16)	
R_{pim}	0.08 (0.673)	0.039 (0.454)	
I/σI	12.6 (1.3)	8.8 (1.5)	
CC1/2	0.999 (0.431)	0.999 (0.363)	
Completeness (%)	98.2 (95.0)	99.5 (99.8)	
Multiplicity	5.5 (4.6)	7.1 (7.2)	
Completeness after anysotropic correction (%)	74.1	83.3	
**Refinement**			
Resolution (Å)	82.93–2.92 (3.08–2.92)	24.96-3.4 (3.49-3.40)	
No. unique reflections	19132	13730	
$R_{work}/R_{free\ (\%)}$	23.9/26.1	23.0/26.8	
Ramachandran favoured (%)	94.0	89.0	
Ramachandran outliers (%)	1.4	2.0	
**R.m.s deviations**			
Bond lengths (Å)	0.017	0.013	
Bond angles (°)	1,97	1,99	

Supplementary Table 1. Data collection and refinement statistics for BasC-nb74 complex.

**Name**	**Sequence (5’-3’)**
BasC S282A mutagenesis	
BasC S282A -F	AACTGGTGACCATTGGTATCCTGATTGCTGTTTTTGGTGGCA
BasC S282A -R	TGCCACCAAAAACAGCAATCAGGATACCAATGGTCACCAGTT
BasC Y236F mutagenesis	
BasC Y236F -F	CCTGTCTATCGTTATGGCAGTGTTTCTGCTGACCAA
BasC Y236F -R	TTGGTCAGCAGAAACACTGCCATAACGATAGACAGG
BasC K154A mutagenesis	
BasC K154A -F	CCTGGCGACGATTCTGGCACTGATCCCGCTGGTT
BasC K154A -R	AACCAGCGGGATCAGTGCCAGAATCGTCGCAGG
BasC K154E mutagenesis	
BasC K154E -F	CCTGGCGACGATTCTGGAGCTGATCCCGCTGGTT
BasC K154E -R	AACCAGCGGGATCAGCTCCAGAATCGTCGCAGG
hAsc-1 S325A mutagenesis	
hAsc-1 S325A -F	CTCCGTGGCTCTGGCAACCTTCGGAGG
hAsc-1 S325A -R	CCTCCGAAGGTTGCCAGAGCCACGGAG
hAsc-1 Y280F mutagenesis	
hAsc-1 Y280F -F	CCCACTGGTGACCTTCGTGGCCACGTTCACCAACATTGCC
hAsc-1 Y280F -R	GGCAATGTTGGTGAACGTGGCCACGAAGGTCACCAGTGGG
hAsc-1 K194A mutagenesis	
hAsc-1 K194A -F	GGACATGTTCACAGGCGGGGCGCTGCTGGCCTTGTCCCTC
hAsc-1 K194A -R	GAGGGACAAGGCCAGCAGCGCCCCGCCTGTGAACATGTCC
hAsc-1 K194E mutagenesis	
hAsc-1 K194E -F	GGACATGTTCACAGGCGGGGAGCTGCTGGCCTTGTCCCTC
hAsc-1 K194E -R	GAGGGACAAGGCCAGCAGCTCCCCGCCTGTGAACATGTCC

Supplementary Table 2. Sequence of used primers

Supplementary References

1. Ma, D. *et al.* Structure and mechanism of a glutamate-GABA antiporter. *Nature* **483,** 632–636 (2012).
2. Krishnamurthy, H. & Gouaux, E. X-ray structures of LeuT in substrate-free outward-open and apo inward-open states. *Nature* **481,** 469–474 (2012).
3. Perez, C. *et al.* Substrate specificity and ion coupling in the Na+/betaine symporter BetP. *EMBO J.* **30,** 1221–9 (2011).
4. Kalayil, S., Schulze, S. & Kühlbrandt, W. Arginine oscillation explains Na+ independence in the substrate/product antiporter CaiT. *Proc. Natl. Acad. Sci. U. S. A.* **110,** 17296–301 (2013).
5. Floden, E. W. *et al.* PSI/TM-Coffee: a web server for fast and accurate multiple sequence alignments of regular and transmembrane proteins using homology extension on reduced databases. *Nucleic Acids Res.* **44,** W339–W343 (2016).
6. Robert, X. & Gouet, P. Deciphering key features in protein structures with the new ENDscript server. *Nucleic Acids Res.* **42,** W320–W324 (2014).
7. Malinauskaite, L. *et al.* A mechanism for intracellular release of Na+ by neurotransmitter/sodium symporters. *Nat. Struct. Mol. Biol.* **21,** 1006–1012 (2014).
