# Supplementary material for: L amino acid transporter structure and molecular bases for the asymmetry of substrate interaction
Source: Nat Commun. 2019 Apr 18;10:1807. doi: 10.1038/s41467-019-09837-z (PMC6472337; doi:10.1038/s41467-019-09837-z)
Supplement: Supplementary file 3 — Reporting Summary [file 41467_2019_9837_MOESM3_ESM.pdf]

nature research

Corresponding author(s): Ekaitz Errasti-Murugarren

Last updated by author(s): Mar 22, 2019

Reporting Summary

Nature Research wishes to improve the reproducibility of the work that we publish. This form provides structure for consistency and transparency in reporting. For further information on Nature Research policies, see Authors & Referees and the Editorial Policy Checklist.

Statistics

For all statistical analyses, confirm that the following items are present in the figure legend, table legend, main text, or Methods section.

n/a	Confirmed	
☐	☒	The exact sample size (*n*) for each experimental group/condition, given as a discrete number and unit of measurement
☐	☒	A statement on whether measurements were taken from distinct samples or whether the same sample was measured repeatedly
☐	☒	The statistical test(s) used AND whether they are one- or two-sided<br>*Only common tests should be described solely by name; describe more complex techniques in the Methods section.*
☒	☐	A description of all covariates tested
☐	☒	A description of any assumptions or corrections, such as tests of normality and adjustment for multiple comparisons
☐	☒	A full description of the statistical parameters including central tendency (e.g. means) or other basic estimates (e.g. regression coefficient) AND variation (e.g. standard deviation) or associated estimates of uncertainty (e.g. confidence intervals)
☐	☒	For null hypothesis testing, the test statistic (e.g. *F*, *t*, *r*) with confidence intervals, effect sizes, degrees of freedom and *P* value noted<br>*Give P values as exact values whenever suitable.*
☒	☐	For Bayesian analysis, information on the choice of priors and Markov chain Monte Carlo settings
☒	☐	For hierarchical and complex designs, identification of the appropriate level for tests and full reporting of outcomes
☒	☐	Estimates of effect sizes (e.g. Cohen's *d*, Pearson's *r*), indicating how they were calculated

Our web collection on statistics for biologists contains articles on many of the points above.

Software and code

Policy information about availability of computer code

Data collection	All software used to collect crystal data was commercially available or open source provided by the corresponding synchrotron
Data analysis	All software used to solve BasC structures was commercially available or open source as indicated in the manuscript

For manuscripts utilizing custom algorithms or software that are central to the research but not yet described in published literature, software must be made available to editors/reviewers. We strongly encourage code deposition in a community repository (e.g. GitHub). See the Nature Research guidelines for submitting code & software for further information.

Data

Policy information about availability of data

All manuscripts must include a data availability statement. This statement should provide the following information, where applicable:

- Accession codes, unique identifiers, or web links for publicly available datasets
- A list of figures that have associated raw data
- A description of any restrictions on data availability

The following is indicated in the main text: Data availability: Atomic coordinates for the crystal structures have been deposited in the Protein Data Bank under accession numbers 6F2G (WT-Nb74 complex) and 6F2W (WT-Nb74 2-AIB co-crystal complex). The source data underlying Figures 2a and b, 3b and d, 4c, 5b, c and d and Supplementary Figures 4b and c, 6 and 10a and b are provided as a Source Data file and deposited in Mendeley. Other data are available from the corresponding author upon reasonable request. PDBs are on hold for publication.

Field-specific reporting

Please select the one below that is the best fit for your research. If you are not sure, read the appropriate sections before making your selection.

☒ Life sciences ☐ Behavioural & social sciences ☐ Ecological, evolutionary & environmental sciences

For a reference copy of the document with all sections, see nature.com/documents/nr-reporting-summary-flat.pdf

Life sciences study design

All studies must disclose on these points even when the disclosure is negative.

Sample size	A minimum of three independent experiments were used in the functional studies (transport activity of BasC reconstituted in proteoliposomes and binding assays of BasC in detergent) as indicated in the manuscript.
Data exclusions	We used GraphPad Prism 7 for non-linear regression and its criteria to eliminate outliers in each independent experiment to fit transport kinetics
Replication	Kinetics of transport activity and dose-response binding assays were performed with a minimum of three independent replicas. Each replica was performed in triplicates or quadruplicates.
Randomization	All samples corresponds to purified BasC protein in different versions (wild type or mutated). To perform independent experiments different protein preparations and reconstitution in proteoliposomes were used.
Blinding	The transport activity of different versions of purifed BasC (wild type and mutants) were studied non-blinded because different assay conditions must be used to measure initial rates of transport of each BasC version.

Reporting for specific materials, systems and methods

We require information from authors about some types of materials, experimental systems and methods used in many studies. Here, indicate whether each material, system or method listed is relevant to your study. If you are not sure if a list item applies to your research, read the appropriate section before selecting a response.

Materials & experimental systems

n/a	Involved in the study
☐	☒ Antibodies
☐	☒ Eukaryotic cell lines
☒	☐ Palaeontology
☒	☐ Animals and other organisms
☒	☐ Human research participants
☒	☐ Clinical data

Methods

n/a	Involved in the study
☒	☐ ChIP-seq
☒	☐ Flow cytometry
☒	☐ MRI-based neuroimaging

Antibodies

Antibodies used	Recombinant Nb74 against the bacterial BasC transporter was generated by the authors
Validation	BasC was crystallized in complex with Nb74 as described in the text

Eukaryotic cell lines

Policy information about cell lines

Cell line source(s)	HeLa cell line to over-express human Asc-1 transporter and test the transport activity
Authentication	HeLa cell line has not been authenticated
Mycoplasma contamination	Routinely mycoplasm is tested in the lab every 15 days
Commonly misidentified lines (See ICLAC register)	Not used
